# Soluble guanylate cyclase crystal clear: 1^st^ crystal structure of the wild-type human heterodimeric sGC catalytic domains and implications for activity

**DOI:** 10.1186/2050-6511-14-S1-O14

**Published:** 2013-08-29

**Authors:** Franziska Seeger, Elsa D Garcin

**Affiliations:** 1 Department of Chemistry Biochemistry, University of Maryland Baltimore County, Baltimore MD 21250, USA

## Background

Soluble guanylate cyclase (sGC) is the key enzyme in the NO-sGC-cGMP signaling cascade crucial in regulating the cardiovascular system. Low output of this system causes hypertension and acute heart failure, which are the leading causes of death globally.

Mammalian sGC is a heterodimer of two homologous subunits (α and β), which contain four domains: an N-terminal regulatory domain (HNOX: Heme Nitric oxide OXygen), an HNOX associated (HNOXA) domain and a coiled-coil (CC) domain important for dimerization, and a

**Figure 1:**
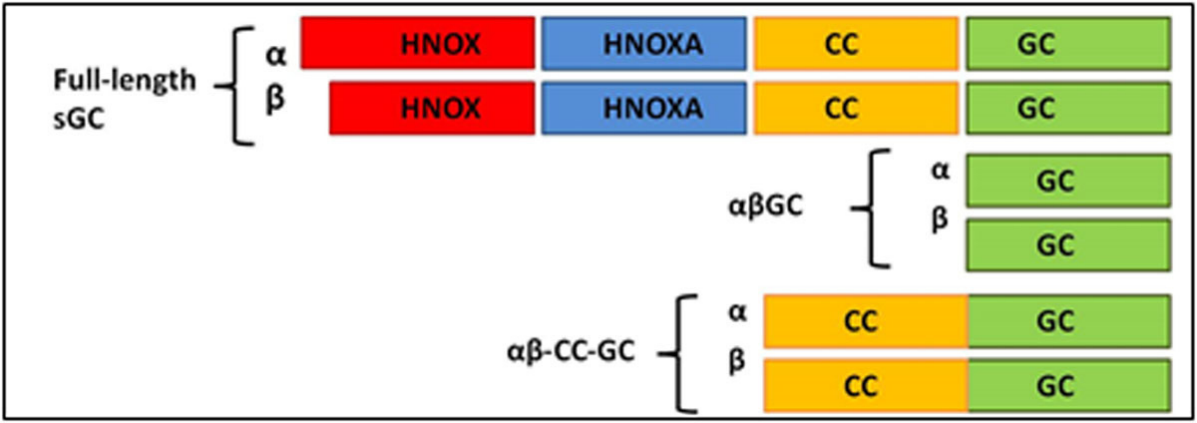
Full-length sGC and C-terminal constructs αβGC and αβCC-GC used in this study

C-terminal catalytic domain (GC) (Figure 1).

The enzyme is basally active, but NO binding to the heme group in the β subunit regulatory domain enhances sGC catalytic output several hundred fold. The molecular mechanism by which the regulatory domain relays the activation signal to the catalytic domain remains elusive. Several studies have highlighted the crucial role of the HNOXA and CC domains for sGC dimerization necessary for catalytic activity [1-9]. Others have shown that the C-terminal GC domains, alone, form catalytically active heterodimers that are inhibited in the presence of the βHNOX regulatory domain [10]. Clearly, more information is needed to elucidate the requirements for sGC activity and activation.

We have established a bacterial overexpression system for truncated constructs of sGC containing the catalytic domains. These constructs can be probed for activity and structurally characterized for conformational changes that may occur during activation.

## Results and discussion

**Figure 2:**
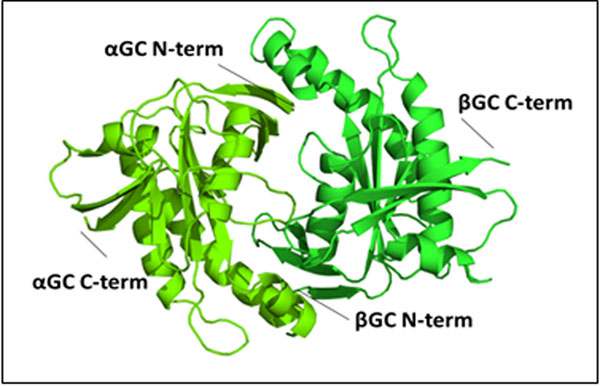
1.9 Å crystal structure of human heterodimeric wild-type catalytic domain of sGC

Here, we report the first crystal structure for the wild type human heterodimeric αβGC catalytic domain to 1.9 Å resolution (Figure 2). Comparison of the heterodimeric αβGC to homodimeric ββGC allows us to identify distinct interactions at the GC dimer interface that can be used to modulate the heterodimer/homodimer equilibrium crucial for activity.

Structural comparisons with adenylate cyclase in its inactive vs. activated state suggest distinct conformational changes that may occur during sGC activation.

Our structural characterization of αβGC combined with activity assay measurements on αβGC, αβCC-GC, and full-length sGC allow us to propose a molecular mechanism for sGC activation. These findings will provide a basis for understanding the mode of action of current and the rational design of novel sGC agonists for application in cardiovascular diseases.
